# Depth of anesthesia, temperature, and postoperative delirium in children and adolescents undergoing cardiac surgery

**DOI:** 10.1186/s12871-023-02102-3

**Published:** 2023-05-02

**Authors:** H. Köditz, A. Drouche, N. Dennhardt, M. Schmidt, M. Schultz, Barbara Schultz

**Affiliations:** 1 Department of Pediatric Cardiology and Intensive Care Medicine, Hannover Medical School, Hannover, Germany; 2 Department of Anesthesiology and Intensive Care Medicine, Hannover Medical School, Hannover, Germany; 3 Medical University of Vienna, Vienna, Austria

**Keywords:** Depth of anesthesia, Body temperature, Delirium, Cardiac surgery, Heart–lung machine, EEG

## Abstract

**Background:**

After pediatric cardiosurgical interventions, postoperative delirium can occur, which can be associated with undesirable consequences during and after the hospital stay. It is therefore important to avoid any factors causing delirium as far as possible. Electroencephalogram (EEG) monitoring can be used during anesthesia to individually adjust dosages of hypnotically acting drugs. It is necessary to gain knowledge about the relationship between intraoperative EEG and postoperative delirium in children.

**Methods:**

In a dataset comprising 89 children (53 male, 36 female; median age: 0.99 (interquartile range: 0.51, 4.89) years) undergoing cardiac surgery involving use of a heart–lung machine, relationships between depth of anesthesia as measured by EEG (EEG index: Narcotrend Index (NI)), sevoflurane dosage, and body temperature were analyzed. A Cornell Assessment of Pediatric Delirium (CAP-D) score ≥ 9 indicated delirium.

**Results:**

The EEG could be used in patients of all age groups for patient monitoring during anesthesia. In the context of induced hypothermia, EEG monitoring supported individually adjusted sevoflurane dosing. The NI was significantly correlated with the body temperature; decreasing temperature was accompanied by a decreasing NI.

A CAP-D score ≥ 9 was documented in 61 patients (68.5%); 28 patients (31.5%) had a CAP-D < 9. Delirious patients with an intubation time ≤ 24 h showed a moderate negative correlation between minimum NI (NI_min_) and CAP-D (rho = -0.41, 95% CI: -0.70 – -0.01, *p* = 0.046), i.e., CAP-D decreased with increasing NI_min_. In the analysis of all patients’ data, NI_min_ and CAP-D showed a weak negative correlation (rho = -0.21, 95% CI: -0.40 – 0.01, *p* = 0.064). On average, the youngest patients had the highest CAP-D scores (*p* = 0.002). Patients with burst suppression / suppression EEG had a longer median intubation time in the intensive care unit than patients without such EEG (*p* = 0.023).

There was no relationship between minimum temperature and CAP-D score.

**Conclusions:**

The EEG can be used to individually adjust sevoflurane dosing during hypothermia. Of the patients extubated within 24 h and classified as delirious, patients with deeper levels of anesthesia had more severe delirium symptoms than patients with lighter levels of anesthesia.

## Background

A heart defect is the most common congenital malformation (1.1% of all live births) [1], and in many of the affected children it requires cardiac surgery at an early age. This often necessitates the use of a heart–lung machine (HLM) during surgery and, after surgery, analgosedation and ventilation in a pediatric or cardiologic intensive care unit. Perioperative mortality for these procedures is low, but a high percentage of children show transient or persistent behavioral problems postoperatively. According to Michel and colleagues (2022), the reported prevalence of delirium is up to 57% in pediatric patients in the intensive care unit after cardiac surgery [2].

According to the Diagnostic and Statistical Manual of Mental Disorders (DSM-5), delirium is a disturbance in attention and awareness which develops over a short time, represents a change from baseline, and tends to fluctuate throughout the day. Diagnosis of delirium requires the presence of at least one additional cognitive disturbance. Furthermore, it is required that the disturbance is not better explained by another neurocognitive disorder, does not occur in the context of a severely reduced level of arousal, and that there is evidence that the disturbance is a direct consequence of another medical condition, substance intoxication or withdrawal, a toxin, or various combinations of causes [3–5].

Postoperative delirium has to be distinguished from emergence delirium [6]. The terms “emergence delirium” and “emergence agitation” have been used interchangeably to describe an agitated state that occurs during emergence from anesthesia and typically is short and largely self-limited [6]. In contrast, the term “postoperative delirium” is used to describe delirium that occurs in patients who have received sedation or general anesthesia, and that may range from postoperative day 0–1 to 5–30 days postoperatively [6].

For delirium screening in children, different scores exist [7, 8]. The Cornell Assessment of Pediatric Delirium (CAP-D) score was developed for patients between 0 and 18 years [9, 10].

Among the risk factors for the occurrence of delirium, a distinction is made between predisposing factors and precipitating factors. Predisposing factors are related to the patient’s baseline status. Precipitating factors are elements that occur throughout the perioperative period and may trigger delirium onset [5, 11]. Nonmodifiable, modifiable, and potentially modifiable factors can be distinguished [12].

Pediatric delirium has been associated with increased length of mechanical ventilation and mortality. However, data on delirium after cardiac surgery in children is scarce [12]. Therefore, it is important to identify factors inducing delirium that arise during the course of anesthesia and critical care, and to develop strategies to minimize or avoid them.

A measure used during cardiac surgery in order to attempt to reduce tissue metabolism and prevent hypoxic damage, particularly in the brain, but also in other tissues, is cooling [13, 14]. Four different levels of systemic hypothermia can be distinguished: mild (35–33 °C), moderate (32–28 °C), deep (27–21 °C) and profound (< 20 °C) [14]. Nevertheless, to date no general consensus on the optimum level of hypothermia for different cardiac surgical interventions in children and adults exists [15–19].

The electroencephalogram (EEG) recorded during anesthesia provides information on the hypnotic component of anesthesia. Increasing doses of hypnotically acting drugs result in a progressive slowing down of the EEG [20]. The EEG is also influenced by body temperature, with a decrease in temperature causing the EEG to slow down [20]. The EEG can be used during heart surgery in children to optimize the dosing of hypnotics during normothermic and hypothermic phases [21]. Other factors that may affect the EEG during anesthesia are hypoxia, hypocapnia, and hypercapnia [20].

Our analysis has the following aims:

1. In a dataset from children with cardiac surgery requiring intraoperative use of a heart lung machine, we wanted to analyze relationships between depth of anesthesia as measured by EEG, sevoflurane dosage, and body temperature.
2. Furthermore, we wanted to analyze the relationship between the intraoperative EEG and the CAP-D score.

## Methods

Ethics approval for the study was obtained from the Ethics Committee of Hannover Medical School, Hannover, Germany (Approval No. 8735-b-os-2019). The study was conducted in accordance with the Declaration of Helsinki. The inclusion criteria for the single-center, prospective, non-randomized study were: patients between 0 and 18 years who had to undergo planned corrective surgery of a congenital heart defect involving the use of HLM, expected postoperative ventilation time of at least 6 h, and expected stay in the intensive care unit (ICU) of more than 24 h. Inclusion was not to be restricted for reasons related to previous illnesses, including those of non-cardiac nature.

Written informed consent was obtained from the patients’ legal caregivers and additionally, if applicable, from the patients themselves, with patients aged 7 to 17 years without mental retardation signing age-appropriate informed consent forms. Data collection took place between December 2019 and April 2021.

The Risk Adjustment for Congenital Heart Surgery (RACHS) score was used for risk classification of the surgical procedures [22]. The patients’ preoperative physical status was documented by means of the American Society of Anesthesiology (ASA) physical status classification system [23].

Etomidate and sufentanil were given as standard medication for induction of anesthesia. Atracurium served as muscle relaxant for intubation. Anesthesia was maintained with sevoflurane and sufentanil. Adjustment of the sevoflurane dose was at the anesthesiologist’s discretion.

Norepinephrine was used during cardiopulmonary bypass to maintain adequate mean arterial pressure. Epinephrine was used before (if necessary), at the end, and after cardiopulmonary bypass in addition to milrinone, and, in rare cases, levosimendan, to support cardiac function and was titrated by using transesophageal echocardiography and central venous oxygenation.

Standard patient monitoring included electrocardiogram, invasive arterial blood pressure, central venous pressure, near-infrared spectroscopy (NIRS), pulse oximetry, capnography, sevoflurane concentration, EEG, nasopharyngeal temperature, rectal or vesical temperature, and urinary output.

In this analysis, documented temperature values and sevoflurane concentrations from the anesthetic record and intraoperative EEG data were analyzed.

The nasopharyngeal temperature was used for data analysis in this study, which focuses on the head/brain. The nasopharyngeal temperature probe was inserted as deep as the distance from the patient’s ear (tragus) to the corner of the mouth. The position of the temperature probe was checked by comparison of the rectal/vesical and the nasopharyngeal temperature, which had to be equal, and during the laryngoscopy for insertion of the transesophageal ECHO probe. If necessary, the position was corrected. The decision on the extent of intraoperative cooling was at the surgeon’s discretion.

The EEGs were recorded intraoperatively as 1-channel recordings with electrode positions on the forehead. The EEG monitor Narcotrend-Compact M (MT MonitorTechnik, Bad Bramstedt, Germany) was used. It performs analyses of the electroencephalograms recorded during anesthesia, and classifies the EEGs. The Narcotrend-Compact M distinguishes the stages A (awake), B_0-2_, C_0-2_, D_0-2_, E_0-2_, F_0_ (burst suppression), and F_1_ (suppression); furthermore, it calculates the Narcotrend Index (NI) (100 to 0). During the first few months of life, children may have a low-differentiated EEG during anesthesia. Therefore, the Narcotrend checks the EEG signal during anesthesia with regard to the level of EEG differentiation. If the EEG is low-differentiated, the stages A (awake), Slow EEG, E_2_, F_0_, and F_1_ are used. The Slow EEG consists of continuous EEG activity without suppression periods. Suppression periods begin to occur in stage E_2_, and the length of suppression periods increases in stages E_2_, F_0_ and F_1_ [21].

Most pediatric cardiac anesthetists at Hannover Medical School use NI for additional information about the depth of hypnosis in combination with age adjusted end-expiratory MAC. This practice is supported by the publications by Dennhardt et al. about reliability of the NI [24] and about the influence of temperature [21]. As the pediatric patients in cardiac anesthesia are often fully relaxed and receive inotropic or vasoactive medication and high doses of opioids, estimating the depth of anesthesia based on clinical findings alone is difficult/impossible. Hypothermia can reduce the level of consciousness and lead to hypnosis. In order to avoid inadequate dosing, using the NI as a tool for additional information seems to be reasonable.

The following temperature, sevoflurane, and EEG values were to be analyzed:

1. The lowest temperature measured for each patient while on the HLM, and the simultaneously recorded sevoflurane concentration and NI.
2. For patients who reached Stage F_1_: the lowest temperature, and the concomitant sevoflurane concentration and NI during stage F_1_.

Furthermore, the lowest temperature and the minimum NI (NI_min_) in each course of anesthesia were evaluated for the time between start of surgery and last suture. The NI_min_ was documented in order to characterize the phase with the lowest EEG index values.

NIRS values were obtained for evaluations from the anesthetic records for the following time points: before induction of anesthesia, before temperature reduction, and at the lowest temperature.

As standard, the patients were transferred to the intensive care unit intubated and ventilated. After the ventilation phase, a delirium interview was to be performed. The parents were to be asked about neurological abnormalities and signs of delirious behavior. If possible, the patients themselves were interviewed. A standardized questionnaire based on the CAP-D score was used [9, 25]. The CAP-D consists of eight questions regarding the patient’s behavior. Per question, up to 4 points can be achieved, resulting in a maximum score of 32 points. A score of ≥ 9 points indicates delirium [25].

Further variables included in the analyses were the anesthesia duration, i.e., the time between start and end of anesthesia, and the incision-suture time as documented in the anesthetic record, i.e., the time between first incision performed by the surgeon and suturing.

In order to show developmental changes in variables, four age groups were used for analyses (≤ 0.5 years, > 0.5 to 1 year, > 1 to 10 years, > 10 years). Children develop particularly quickly within the first year of life. Therefore, a subdivision within the first year of life seemed reasonable. The limit of 10 years was chosen as, beyond the age of 10, the transition to puberty and adolescence takes place.

### Statistics

The data was analyzed using SAS (SAS Institute, Cary, USA), version 9.3 and JASP (JASP Team 2022), version 0.16.4. Data was checked for normal distribution. Mean and standard deviations are provided for variables with normal distribution; median and interquartile range (IQR) are used for variables without normal distribution. If normal distribution was not confirmed, a non-parametric test was used. The following statistical methods were used: chi-squared test, independent samples t-test, paired samples t-test, Mann–Whitney test, regression analysis, and Kruskal–Wallis test. For correlations, Spearman’s correlation coefficient rho was used. Strength of correlation is described based on suggestions by Schober et al. [26] (rho 0.00–0.09: negligible correlation, rho 0.10–0.39: weak correlation, rho 0.40–0.69: moderate correlation, rho 0.70–0.89: strong correlation, rho 0.90–1.00: very strong correlation). The significance threshold was assumed to be *p* < 0.05.

## Results

Data from 89 patients (53 male, 36 female) was evaluated. The median age was 0.99 (interquartile range: 0.51, 4.89) years. The surgical interventions can be grouped according to the following anomalies: valvular defects (26 patients), septal defects (26 patients), single ventricle (14 patients), tetralogy of Fallot (12 patients), atrioventricular canal defects (6 patients), and others (5 patients) (Table 1).

**Table 1:** Demographic data, cardiac anomalies, and clinical data (Data is expressed as *n* (%), median (IQR), or mean ± standard deviation.)

	CAP-D ≥ 9	CAP-D < 9	*P*
**Demographic data**			
Male / female	35 (57.4%) / 26 (42.6%)	18 (64.3%) / 10 (35.7%)	0.644
Age (years)	0.70 (0.48, 2.86)	3.64 (0.67, 9.20)	0.015
Weight (kg)	7.30 (5.50, 12.40)	16.33 (6.67, 28.70)	0.015
Height (cm)	67.00 (63.00, 89.00)	100.00 (65.00, 131.75)	0.024
**Cardiac anomalies**			
Valvular defect	17 (27.9%)	9 (32.1%)	
Septal defect	21 (34.4%)	5 (17.9%)	
Single ventricle	9 (14.8%)	5 (17.9%)	0.402
Tetralogy of Fallot	7 (11.5%)	5 (17.9%)	
Atrioventricular canal defect	5 (8.2%)	1 (3.6%)	
Other	2 (3.3%)	3 (10.7%)	
**Clinical data**			
Anesthesia duration (hours)	6.65 (5.82, 7.86)	6.73 (5.98, 7.36)	0.781
Incision-suture time (hours)	4.86 (3.90, 6.04)	5.08 (4.25, 5.93)	0.606
Duration on heart-lung machine (hours)	3.17 ± 1.00	3.05 ± 1.00	0.603
Sufentanil (µg/kg/hour anesthesia duration)	0.98 (0.92, 1.02)	0.97 (0.93, 0.99)	0.356
Norepinephrine (µg/kg/min anesthesia duration)	0.022 (0.009, 0.038)	0.022 (0.006, 0.038)	0.945
Epinephrine (µg/kg/min anesthesia duration)	0.014 (0.007, 0.027)	0.006 (0.000, 0.024)	0.039

*CAP-D* Cornell Assessment of Pediatric Delirium

In 61 patients (68.5%), a CAP-D score of ≥ 9 indicating delirium was documented; in 28 patients (31.5%), the CAP-D score was below 9. Table 1 shows that patients with CAP-D ≥ 9 and patients with CAP-D < 9 did not differ significantly with regard to gender, whereas they did with regard to age, weight, and height. The types of cardiac anomalies, the anesthesia duration, the incision-suture time, the duration on the heart–lung machine, the sufentanil dosage, and the norepinephrine dosage were not significantly different between patients with CAP-D ≥ 9 and patients with CAP-D < 9 (Table 1). Patients with CAP-D ≥ 9 received a significantly higher epinephrine dosage than patients with CAP-D < 9.

### Example of a course of anesthesia

**Fig. 1:**
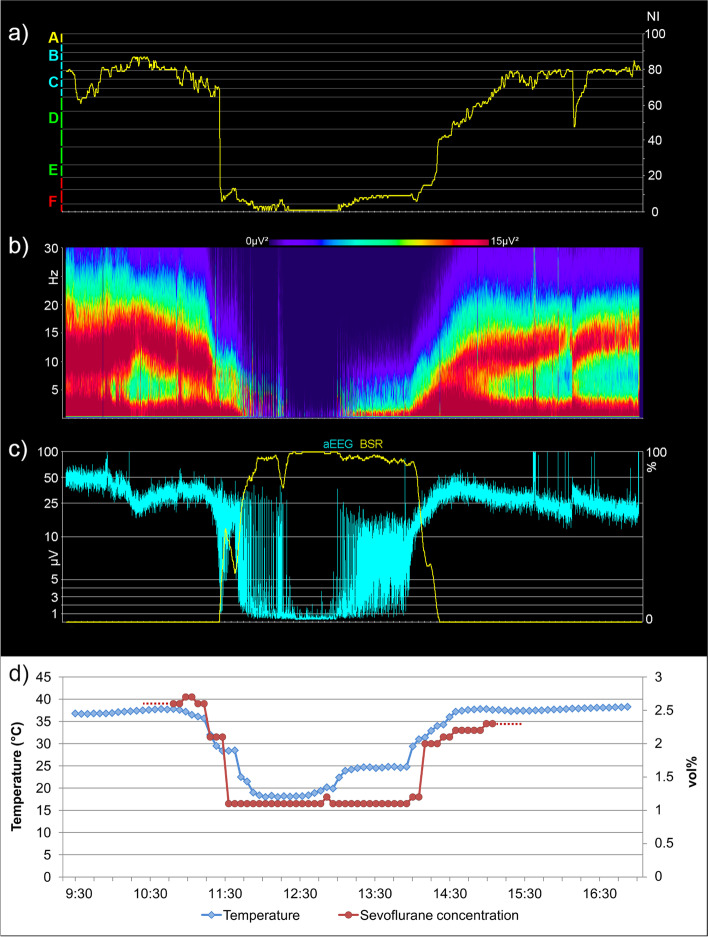
Course of anesthesia in an 11-month-old child. **a)** Cerebrogram **b)** Density spectral array (DSA) **c)** Amplitude-integrated EEG (aEEG) and burst suppression ratio (BSR) **d)** Temperature and sevoflurane concentration

The EEG was part of the patient monitoring during anesthesia. As an example, Fig. 1 shows the trend of the EEG index/stages (cerebrogram), the density spectral array (DSA), the amplitude-integrated EEG (aEEG), and the trends of temperature and sevoflurane concentration from a course of anesthesia in an 11-month-old child. In the beginning and in the end, mainly C and B stages occurred, indicating a moderate to light level of hypnosis. Between 11:30 and 14:20, during the HLM period, EEG stages in the E_2_ to F_1_ range occurred. The temperature was decreased during the HLM period. It was below 30 °C at 11:30, below 20 °C from 11:50 to 12:45, and just below 25 °C from 13:05 to 13:55. Increasing gradually thereafter, a temperature above 35 °C was reached at 14:30. The sevoflurane concentration was reduced during the cooling period.

### Relationship between temperature, sevoflurane concentration, and NI

During the HLM period, there was a standard drop in temperature, with the degree of the temperature drop varying between individuals.

When the end-tidal sevoflurane concentration was compared before cooling and at the lowest temperature, most patients (74.7%) showed a decrease in end-tidal sevoflurane concentration, and about a quarter of patients (25.3%) showed an increase or the same concentration. The mean decrease of the end-tidal sevoflurane concentration was greater than the increase (-1.0 ± 0.6 vs. 0.3 ± 0.3 vol%; mean percentage decrease/increase: -38.4 ± 21.1 vs. 15.5 ± 13.8%; patients with no decrease/increase not included). In the majority of patients (89.2%), the NI showed a decrease between the time before cooling and the time at the lowest temperature; in 10.8% of patients the NI increased or was the same. The mean decrease of the NI was greater than the increase (-35.5 ± 20.1 vs. 3.6 ± 2.9 points on the NI scale (mean percentage decrease/increase: -59.0 ± 30.5 vs. 7.7 ± 6.5%); patients with no decrease/increase not included).

**Fig. 2:**
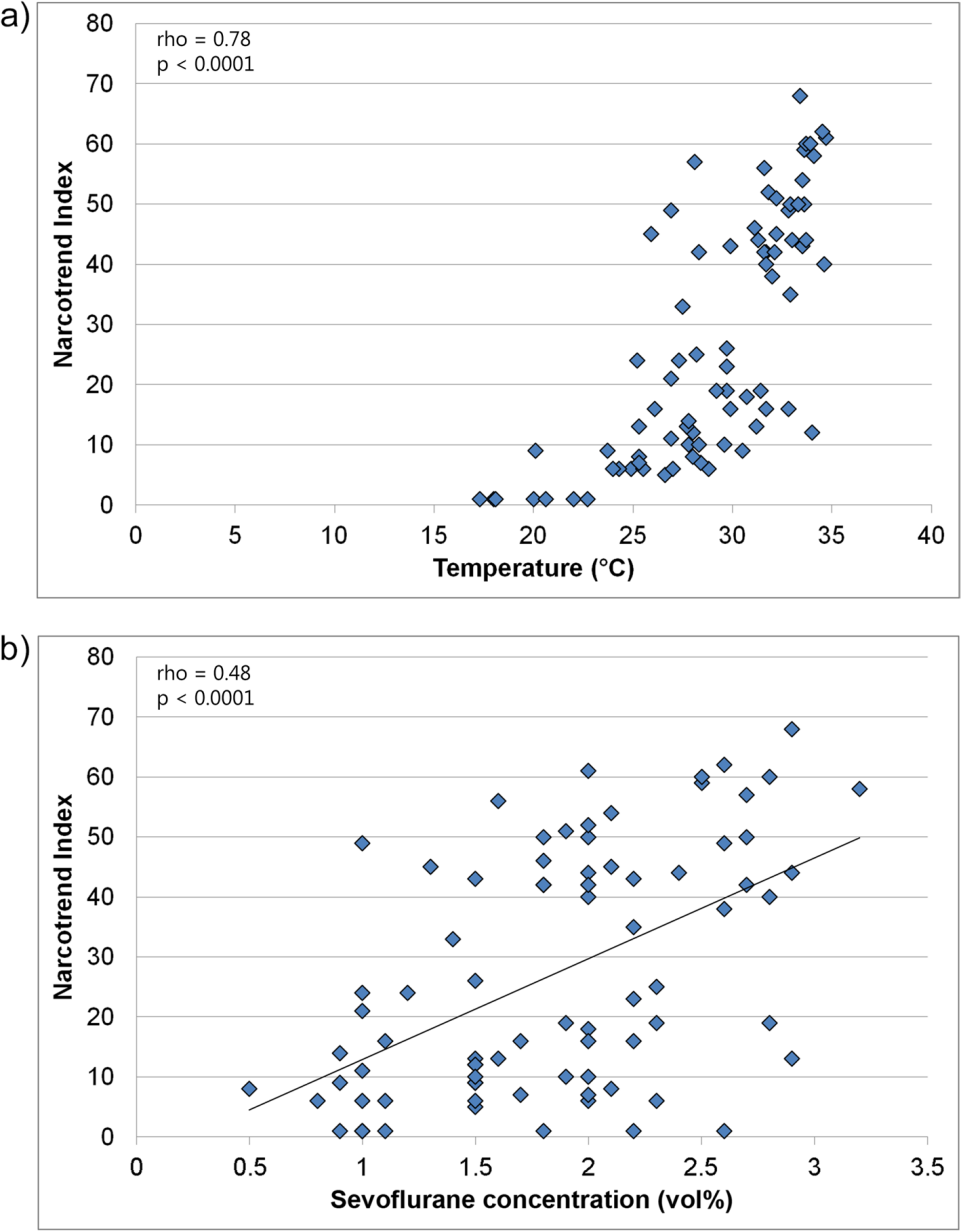
Relation between **a)** Narcotrend Index and temperature **b)** Narcotrend Index and sevoflurane concentration for the time point with the lowest temperature during the HLM period

To analyze the relationship between the lowest temperature, the sevoflurane concentration, and the NI, for each patient, the lowest temperature during the HLM period, the concomitant sevoflurane concentration, and the NI were evaluated. Figure 2a shows that the NI decreased significantly with decreasing temperature. There was a strong correlation between NI and temperature (rho = 0.78, 95% confidence interval (CI): 0.67 – 0.85, *p* < 0.0001). Figure 2b shows that the sevoflurane concentration decreased with decreasing NI. The correlation between NI and sevoflurane was moderate (rho = 0.48, 95% CI: 0.29 – 0.64, *p* < 0.0001). The sevoflurane concentration at the lowest temperature was between 0.5 and 3.2 vol%.

Patients with and without delirium did not differ significantly with respect to the sevoflurane concentrations at the two points in time studied in the course of anesthesia, before cooling (2.6 ± 0.4 vs 2.5 ± 0.3 vol%, *p* = 0.2635) and at the lowest temperature (1.9 ± 0.6 vs 1.9 ± 0.7 vol%, *p* = 0.9600).

### Temperature and sevoflurane concentration in EEG stage F_1_

**Fig. 3:**
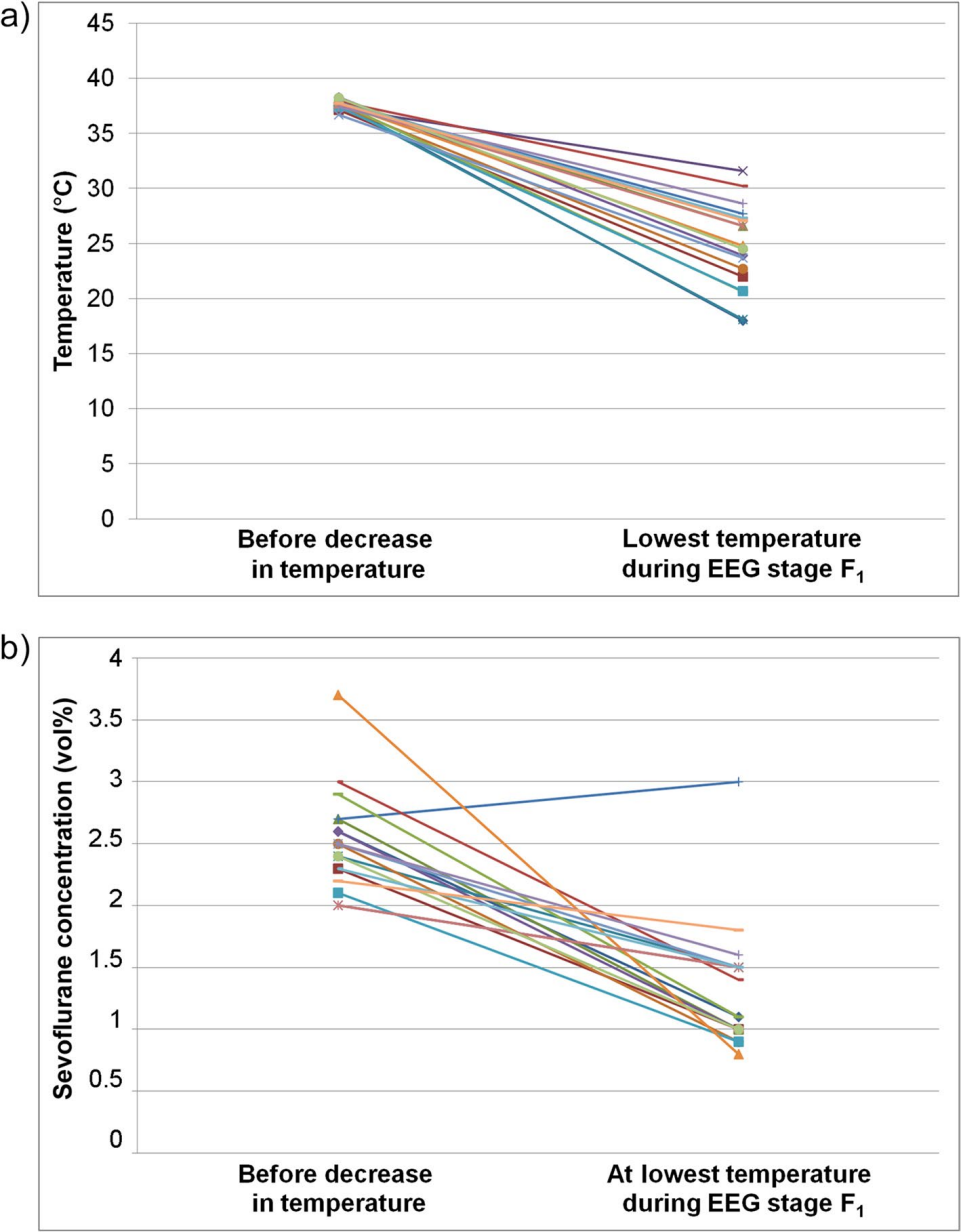
**a)** Temperature and **b)** sevoflurane concentration before cooling and in EEG stage F_1_ (suppression) during cooling

EEG stage F_1_ was reached by 18 patients during hypothermia. Figure 3 shows the temperature and the sevoflurane concentration before cooling, and at the lowest temperature in stage F_1_ (EEG suppression) during the HLM period. The changes between the two time points were significant for both variables (temperature: *p* < 0.0001; sevoflurane concentration: *p* < 0.0001). In stage F_1_, the temperatures ranged from 18 to 31.6 °C in the 18 patients; the range of the sevoflurane concentrations was between 0.8 and 1.8 vol% for 17 of the 18 patients; in one patient the sevoflurane concentration was 3.0 vol%. Figure 3b shows that the reduction of sevoflurane doses did not follow a predefined pattern, but was adapted to the respective situation; for example, the patient with the highest sevoflurane dose before cooling had the lowest sevoflurane concentration at the lowest temperature. In one patient, there was no reduction, but an increase in dose. In this patient, the anesthesiologist decided to administer sevoflurane at a comparably high concentration (2.9 vol%) for most of the time during the operation, with a plateau in E/F stages.

### Minimum EEG index, minimum temperature, and CAP-D score

In the following analyses, the whole intraoperative time between start of surgery and last suture was considered.

#### *Relationship between NI*_*min*_* and CAP-D score*

The minimum Narcotrend Index (NI_min_) for the time between start of surgery and last suture showed a weak correlation with the CAP-D score (rho = -0.21, 95% CI: -0.40 – 0.01, *p* = 0.064). The CAP-D score decreased with increasing NI_min_.

**Fig. 4:**
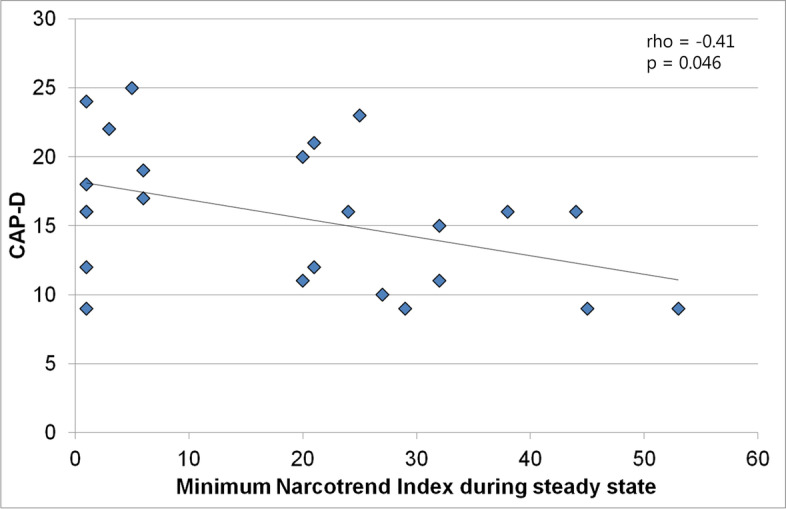
Minimum Narcotrend Index (NI_min_) and CAP-D score in patients who had a CAP-D ≥ 9 and an intubation time ≤ 24 h

A separate evaluation was performed for patients who had an intubation time ≤ 24 h and who were classified as delirious according to the CAP-D score (CAP-D ≥ 9). The time interval of ≤ 24 h was chosen to minimize possible influences related to the stay in the intensive care unit. In these patients, there was a moderate negative correlation between NI_min_ and CAP-D (rho = -0.41, 95% CI: -0.70 – -0.01, *p* = 0.046). Figure 4 shows that higher CAP-D values (≥ 17) were present for NI_min_ values ≤ 25, but did not occur with NI_min_ > 25, i.e., patients with deeper levels of anesthesia experienced more severe symptoms of delirium than patients with lighter levels of anesthesia. There was a weak negative correlation between patient age and CAP-D in the patients included in this analysis (rho = -0.31, 95% CI: -0.62 – 0.08, *p* = 0.116).

#### Minimum temperature

The minimum temperature during the time between start of surgery and last suture was identical to the minimum temperature while on the HLM. The minimum temperature showed a negligible relationship with the CAP-D score (rho = -0.02, 95% CI: -0.23 – 0.19, *p* = 0.841). The nasopharyngeal temperature (minimum temperature) correlated very strongly with the minimum rectal/vesical temperature (rho = 0.98, 95% CI: 0.97 – 0.99, *p* < 0.0001).

#### RACHS score

The RACHS score showed a weak correlation with the minimum temperature (rho = -0.16, 95% CI: -0.36 – 0.05, *p* = 0.128) documented intraoperatively or the CAP-D score (rho = -0.13, 95% CI: -0.33 – 0.08, *p* = 0.237). Out of the 89 patients in the study, 86 had a RACHS score of 2 or 3.

#### Duration of intubation

The group of patients with burst suppression / suppression EEG had a longer intubation time in the ICU than the group without burst suppression / suppression EEG (*p* = 0.023). In the group with a burst suppression / suppression EEG, the median intubation time was 25.7 (9.8, 82.8) hours; in the group without burst suppression / suppression EEG, the median intubation time was 12.9 (8.5, 26.2) hours.

### Near-infrared spectroscopy (NIRS)

There was a negligible correlation between the CAP-D and the NIRS values noted a) before induction of anesthesia (rho = -0.09, 95% CI: -0.30 – 0.13, *p* = 0.430), b) before temperature reduction (rho = -0.09, 95% CI: -0.29 – 0.12, *p* = 0.414), and c) at the lowest temperature (rho = -0.07, 95% CI: -0.27 – 0.14, *p* = 0.523).

### Evaluation of parameters in four age groups

**Fig. 5:**
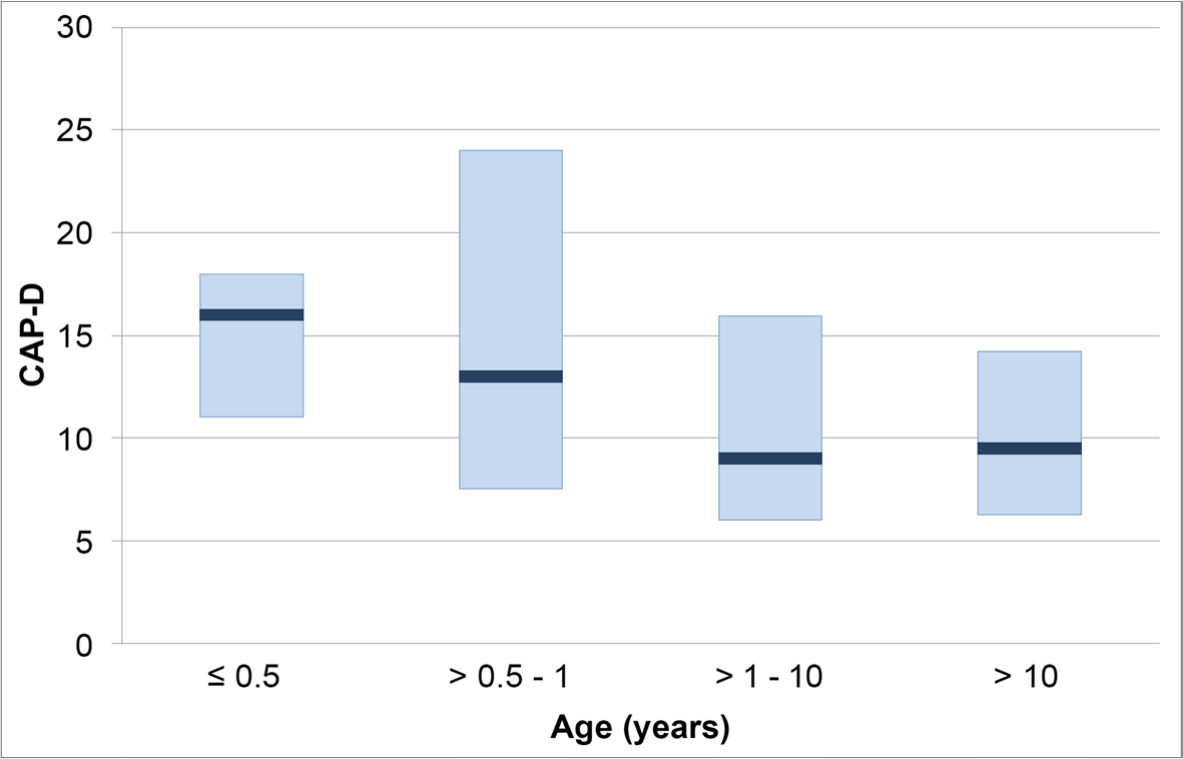
CAP-D score across four age groups. Dark blue bars: 50^th^ percentile (median), lower and upper margin of light blue boxes: 25^th^ and 75^th^ percentile

For each of the six variables NI_min_, intubation time in the intensive care unit, CAP-D, incision-suture time, minimum temperature, and sevoflurane concentration at minimum temperature, the median and the IQR were calculated for four age groups (Table 2). Children within the first year of life had the lowest NI_min_, the shortest incision-suture time, and the highest CAP-D score. The sevoflurane concentration was weakly correlated with age. The minimum temperature showed a negligible relationship with age. Figure 5 shows the CAP-D scores for the four age groups.

**Table 2:** NI_min_, intubation time in the intensive care unit, CAP-D score, incision-suture time, sevoflurane concentration at minimum temperature, and minimum temperature in the operating room across four age groups (median (IQR), Spearman's correlation coefficient rho, 95% CI, and the *p* value for the correlation of each variable with age)

Age (years)	N	NI_min_	Intubation time (hours)	CAP-D	Incision-suture-time (hours)	Sevoflurane concentration (vol%) at minimum temperature	Minimum temperature (°C)
≤ 0.5	22	5 (1.0, 10.8)	78.2 (29.2, 136.5)	16 (11.0, 18.0)	4.1 (3.8, 4.9)	1.8 (1.5, 2.0)	31.5 (27.9, 32.7)
> 0.5 – 1	24	6 (1.0, 20.0)	29.8 (15.2, 70.3)	13 (7.5, 24.0)	5.0 (4.2, 5.9)	1.9 (1.1, 2.0)	28.8 (24.8, 31.6)
> 1 – 10	29	21 (3.5, 32.0)	11.2 (7.2, 24.8)	9 (6.0, 16.0)	5.3 (4.6, 6.1)	1.8 (1.5, 2.1)	28.3 (26.9, 32.0)
> 10	14	27.5 (20.3, 40.8)	10.8 (7.7, 17.9)	9.5 (6.3, 14.3)	5.3 (4.1, 6.4)	2.3 (2.2, 2.7)	31.8 (28.6, 33.4)
rho 95% CI		0.50 0.32 – 0.65	-0.55 -0.68 – -0.39	0.32 -0.49 – -0.12	0.26 0.05 – 0.44	0.31 0.09 – 0.49	0.07 -0.13 – 0.27
*p*		< 0.0001	< 0.0001	0.002	0.014	0.006	0.53

*CAP-D* Cornell Assessment of Pediatric Delirium, *CI* Confidence interval, *IQR* Interquartile range, *NI*_*min*_ Minimum Narcotrend Index

The median ASA score was not significantly different across the four age groups; in all four groups the median ASA score was 3 (3, 3) (*p* = 0.955).

## Discussion

This paper describes anesthesia that included phases of hypothermia. The EEG was available as part of standard patient monitoring during anesthesia. The results showed that the EEG index NI was significantly correlated with the temperature. A decreasing temperature was accompanied by a decreasing NI. The analyses revealed a correlation between depth of anesthesia and delirium as measured by the CAP-D. The minimum temperature did not display a relationship with the CAP-D score. The youngest patients had the highest CAP-D scores on average.

The results of the study show that EEG was actively used by the anesthesiologists during the surgical interventions when dosing sevoflurane. In addition to the EEG index, e.g., the DSA and the aEEG were available, which could be used according to the anesthesiologist’s preferences.

The anesthesiologists could use the EEG as a guide to avoid a burst suppression / suppression EEG as an expression of very deep anesthesia if very deep anesthesia was not intended. In deep hypothermia, burst suppression / suppression may occur due to temperature. Under these conditions, to avoid further enhancing a temperature-related burst suppression / suppression EEG, the sevoflurane concentration could be reduced. In situations where the EEG indicated unintended very light stages of anesthesia, the sevoflurane concentration could be increased.

In our clinical experience, the use of EEG leads to reduced dosages during hypothermia; the administration of unnecessarily high dosages can be avoided. Without a reduction in the sevoflurane dosage, a permanent suppression over longer periods of time would have been expected.

In the group of patients who were extubated within 24 h and classified as delirious, patients with lighter levels of anesthesia had less severe delirium symptoms than patients with deeper levels of anesthesia. In Fig. 4, although there are no CAP-D values in the NI_min_ range from 7 to 19, it can be seen that high CAP-D values were associated with NI_min_ values of deeper anesthesia and not with NI_min_ values of lighter anesthesia. In this analysis, deeper levels of anesthesia were characterized by an NI_min_ ≤ 25. An NI of 25 is related to stage E_1_, which is characterized by continuous delta activity (0.5 – 3.5 Hz), and which is located above stage E_2_, the transition to the burst suppression pattern.

In adult patients, several studies have shown an association of intraoperative burst suppression patterns with postoperative delirium [27, 28]. In a systematic review and meta-analysis, MacKenzie and colleagues (2018) concluded that processed EEG-guided anesthesia was associated with a decrease in postoperative delirium [27]. The authors of a Cochrane analysis from 2018 came to a similar conclusion [28]. In a guideline on postoperative delirium, the European Society of Anaesthesiology recommends avoiding unnecessarily deep anesthesia, often reaching burst suppression in elderly patients [5]. According to a consensus statement on the role of neuromonitoring on perioperative outcomes, it is advisable to avoid unintended burst suppression during anesthesia [29]. In children, there are few studies that analyze intraoperative dosages of hypnotic drugs and postoperative delirium symptoms, taking into account the intraoperative EEG [30–32]. A single-center study on intraoperative burst suppression and emergence delirium in children did not find a significant association of the occurrence of burst suppression and emergence delirium [32]. Emergence agitation occurs usually within the first 30 min after anesthesia, and is characterized by combativeness, excitation, disorientation, and inconsolability [33]. In a single- and in a multi-center study in children up to 37 or 36 months of age, respectively, isoelectric events during anesthesia were not associated with emergence behavior [34, 35]. After anesthesia with the inhalation anesthetic sevoflurane, which was given in our study, emergence delirium occurs particularly frequently in children [36]. In a study of adult patients with major abdominal surgery, one intraoperative factor that was related with the occurrence of delirium was the minimum alveolar concentration (MAC) of sevoflurane, which was higher in patients with postoperative delirium compared to patients without postoperative delirium (*p* = 0.03) [37].

It is quite conceivable that the incidence of postoperative delirium in patients may be different when anesthetic agents other than sevoflurane are used. For example, a recent reanalysis of a study in older patients found that desflurane was associated with a higher risk of developing postoperative delirium as compared to propofol or sevoflurane, even though prolonged intraoperative burst suppression activity occurred with propofol [38]. In another study, older patients with elective lower extremity orthopedic surgery under spinal anesthesia were randomized to receive dexmedetomidine or propofol sedation. Patients with dexmedetomidine sedation had a lower incidence of delirium than patients with propofol sedation [39].

The European Society of Anesthesiology recommends continuous infusion of opioids during anesthesia as one measure among others to prevent postoperative delirium [5]. The children in our study received sufentanil as continuous infusion. The dosages of patients with delirium and patients without delirium did not differ significantly.

In pediatric intensive care patients, vasopressor administration has been identified as a factor associated with the occurrence of delirium [40–43]. In a study of adult patients, both maximum intraoperative and maximum postoperative norepinephrine dosages were higher in patients with delirium than in patients without delirium [44]. In our study, norepinephrine dosages were not significantly different between patients with and without delirium. Patients with delirium received higher dosages of epinephrine. Whether this may have contributed to the development of delirium remains speculative.

In our study, a CAP-D score of ≥ 9 was documented in 68.5% of the patients. Among the patients with an intubation time ≤ 24 h, the incidence of a CAP-D score ≥ 9 was 55.0%. An expected postoperative ventilation time < 6 h was an exclusion criterion in our study. Patel et al. reported a delirium incidence of 49% in children after cardiac surgery, but 36% of children were extubated while still in the operating room [40]. The exclusion of patients with an expected postoperative ventilation time < 6 h may have influenced the delirium incidence in our study.

Considering the potential consequences of delirium, such as increased length of mechanical ventilation, hospital costs, and even mortality [12], strategies for the prevention of pediatric delirium need to be optimized [45, 46]. In order to avoid inappropriate dosing of hypnotically acting drugs, which is a potential modifiable risk factor for delirium, intraoperative EEG monitoring should be part of the measures to optimize the treatment of children undergoing cardiac surgery.

It is known from studies on adult patients that EEG changes in response to hypothermia differ interindividually. In a study by Coselli et al. investigating 56 patients who required circulatory arrest as part of an operation on the ascending aortic arch or the aortic valve, the patients achieved electrical silence, i.e., complete EEG suppression, at a nasopharyngeal temperature between 10.1 and 24.1 °C. Esophageal and rectal temperature measurements also revealed wide temperature ranges. The authors concluded that monitoring the EEG to identify electrocerebral silence is a safe, consistent, and objective method of determining the appropriate level of hypothermia [47].

In this study, there was no relationship between the minimum temperature on cardiopulmonary bypass and the CAP-D. During cardiac surgery, hypothermia is used in a preventive manner, contrary to the use of hypothermia in the context of resuscitation, where it is applied after resuscitation. In neonates who have experienced cerebral hypoxia during delivery [48] and in pediatric patients of different ages after resuscitation [49], hypothermia has been shown to improve patient outcome. A number of authors have addressed the rewarming rate at the end of the HLM phase [50–53]. In some studies, the relationship between rewarming rate and postoperative cognitive function in adult patients was examined; these studies provided different results, the incidence of postoperative delirium was not examined [54–57].

In our study, the median CAP-D score was highest in the group of patients aged up to 0.5 years, and second-highest in the group > 0.5 to 1 year. Age < 2 years has been described as a risk factor for delirium after cardiopulmonary bypass [58].

Out of the 4 age groups, the patients in the two youngest age groups had the lowest NI_min_ (Table 2) in the time period from start of surgery to suturing; both median values were in the burst suppression range. Interestingly, however, the median lowest intraoperative temperatures in these children were similar to those in the older children, and the median sevoflurane concentration was not higher than in the older patients. The particularly low EEG index values may indicate a particular sensitivity in the two youngest age groups with regard to sevoflurane dosing or cooling.

It remains to be considered whether burst suppression EEG patterns can be avoided in very young children under the circumstances of cardiac surgery and whether this improves outcome with regard to the incidence of postoperative delirium.

### Limitations

Our analysis considered temperature, NI, sevoflurane concentration, and near-infrared spectroscopy. However, the development of delirium is a multifactorial process.

It might be valuable to analyze the duration of low NI and hypothermia for defined time periods with respect to the occurrence of delirium. We have chosen to analyze the lowest values of NI and temperature as extreme values. Typically, there are variations in NI and temperature over time.

Sufentanil has been shown to cause a dose-dependent slowing of the EEG [59, 60]. Investigation of the effect of sufentanil on EEG was not an aim of the study. Patients with a CAP-D ≥ 9 and patients with a CAP-D < 9 did not differ significantly with respect to sufentanil dosage.

Possible correlations between the EEG and the administration of vasoactive substances were not investigated. The investigation of such effects was beyond the scope of this study.

The heterogeneity in target temperature required for the different surgical procedures resulted in high variability in NI and sevoflurane dose. Furthermore, according to the Narcotrend indicating very deep anesthesia or burst suppression, administration of sevoflurane could still have been reduced more during hypothermia in some cases.

The CAP-D was collected with the participation of the parents or the patients themselves. The high number of contributors harbors the risk of inconsistent evaluation. However, all interviews were performed by the same investigator. Furthermore, some authors take the view that the parents’ assessment with regard to delirium symptoms is a helpful contribution in practice [61].

## Conclusions

It was shown that the EEG can be used to individually adjust sevoflurane dosing during hypothermia. In the group of patients who were extubated within 24 h and classified as delirious, patients with deeper levels of anesthesia had more severe delirium symptoms than patients with lighter levels of anesthesia.

## Data Availability

The datasets generated during and/or analyzed during the current study are available from the corresponding author on reasonable request.

## Abbreviations

aEEG: Amplitude-integrated EEG
ASA: American Society of Anesthesiologists
BSR: Burst suppression ratio
CAP-D: Cornell Assessment of Pediatric Delirium
CI: Confidence interval
DSA: Density spectral array
DSM-5: Diagnostic and Statistical Manual of Mental Disorders, 5^th^ edition
EEG: Electroencephalogram
HLM: Heart–lung machine
ICU: Intensive care unit
IQR: Interquartile range
JASP: Jeffreys’s Amazing Statistics Program
NI: Narcotrend Index
NI_min_: Minimum Narcotrend Index
NIRS: Near-infrared spectroscopy
MAC: Minimum alveolar concentration
RACHS: Risk Adjustment for Congenital Heart Surgery
SAS: Statistical Analysis System
