# Correction: Depth of anesthesia, temperature, and postoperative delirium in children and adolescents undergoing cardiac surgery

**DOI:** 10.1186/s12871-023-02272-0

**Published:** 2023-09-15

**Authors:** H. Köditz, A. Drouche, N. Dennhardt, M. Schmidt, M. Schultz, Barbara Schultz

**Affiliations:** 1 Department of Pediatric Cardiology and Intensive Care Medicine, Hannover Medical School, Hannover, Germany; 2 Department of Anesthesiology and Intensive Care Medicine, Hannover Medical School, Hannover, Germany; 3 Medical University of Vienna, Vienna, Austria

**Correction: BMC Anesthesiol 23, 148 (2023)**

10.1186/s12871-023-02102-3

Following publication of the original article [1] it was brought to our attention that the familial ties of two of the authors, Barbara Schultz and M. Schultz, to Narcotrend were not fully declared at submission. The correct Competing Interests statement is:

“HK, AD, ND, and MSchm declare that they do not have competing interests. BS and MSchu state a family relationship with Arthur Schultz. He is the inventor and owner of patents which are used as basis of Narcotrend products and the coordinator of Narcotrend projects. BS was involved in EEG evaluations of depth of anesthesia for Narcotrend.“
